# Amelioration of dextran sulfate sodium-induced colitis by autoinducer-2-deficient *Lactiplantibacillus plantarum* is mediated by anti-inflammatory effects and alleviation of dysbiosis of the gut microbiota

**DOI:** 10.3389/fmicb.2022.1013586

**Published:** 2022-09-14

**Authors:** Yilin Qian, Lei Ma, Mingyong Zeng, Zunying Liu

**Affiliations:** ^1^College of Food Science and Engineering, Ocean University of China, Qingdao, China; ^2^Qingdao Engineering Research Center for Preservation Technology of Marine Foods, Qingdao, China

**Keywords:** *Lactiplantibacillus plantarum*, DSS, AI-2 deficient mutant, gut microbiota, anti-inflammatory

## Abstract

Lactic acid bacteria (LAB) attenuate dextran sulfate sodium (DSS)-induced colitis in mice by restoring gut flora homeostasis and modulating the immune response. Because synchronous behavior can be controlled by autoinducer-2 (AI-2)/LuxS-mediated quorum sensing, the Caco-2 cell model and DSS-induced model in C57BL/6 mice were used to explore the unknown effects of these communications involving AI-2 among various intestinal symbiotic species. The results of the cell viability and lactate dehydrogenase leakage assays indicated that the tested strains (the wild-type strains and AI-2-deficient mutants) were characterized by equal cytoprotection from hydrogen peroxide-induced injury independently of AI-2. The results of the assays of multiple indicators and proinflammatory cytokines characteristic for the symptoms of colitis in mice showed that oral administration of AI-2-deficient mutants for 7 days was more effective in ameliorating inflammation than the treatment with the wild-type strains. The treatment with AI-2-deficient mutants enriched potential probiotics (e.g., Lactobacillaceae) and controlled the proliferation of potentially harmful bacteria (e.g., Helicobacteraceae) to achieve the transformation of intestinal flora. These mutants regulated short-chain fatty acids and the intestinal epithelial barrier, thereby promoting the maintenance of relatively favorable intestinal homeostasis. These results demonstrated that the AI-2-deficient mutants provided a more pronounced ameliorative effect on colitis in a mouse model, suggesting that the background of the LAB effect is associated with the alterations in colonic flora induced by AI-2.

## Introduction

Inflammatory bowel disease (IBD), including Crohn’s disease and ulcerative colitis, is the second most common inflammatory disease after rheumatoid arthritis (Neurath, 2014). Extraintestinal manifestations of inflammatory enteritis are particularly severe and debilitating and have high prevalence, requiring expedient surgery; IBD is known as pathology with tremendous socioeconomic consequences (Petrella, 2016). The complexity of IBD pathogenesis has been extensively studied and is associated with abnormal ecology of the gut microbiome or deficiency of immune sensitivity, which may even be inherited (Celiberto et al., 2017). Thus, the intestinal microbiome was proposed to mediate gastrointestinal mucosal homeostasis and regulate IBD in humans under ecologically dysregulated conditions (Carlsson et al., 2013; Martens et al., 2018; Xu et al., 2021). Therefore, more effective and safer approaches are being developed to prevent or alleviate the development of IBD.

Lactic acid bacteria (LAB) ameliorate dextran sulfate sodium (DSS)-induced colitis by restoring homeostasis of intestinal flora, and modulation of the immune response by LAB has been reported (Rodríguez-Nogales et al., 2017; Sun et al., 2020; Wang et al., 2020). Intestinal probiotic preparations targeting the regulation of intestinal microorganisms have attracted considerable attention for the treatment of colitis, unlike traditional drugs (e.g., aminosalicylates), because these preparations do not cause side effects. Specific approaches underlying the protective effect of LAB in the intestine are presumed to be multifactorial, involving fermentation products, the production of bioactive or metabolic compounds and competition for the mucosal sites (Wine et al., 2010; Rodríguez-Nogales et al., 2017). However, few studies have evaluated the synchronous communication between LAB and intestinal flora, and the effect of regulating their quorum behavior on the recovery of intestinal inflammation.

Bacteria synthesize, release and sense the signal compounds considered autoinducers based on bacterial cell number, and these compounds regulate the group behaviors in a process called quorum sensing (QS). These signals are generally species-specific, and autoinducer-2 (AI-2) is the only known signal that appears to be shared by the entire bacterial world (Thompson et al., 2015). Li J. et al. (2019) demonstrated an increase in AI-2 in the intestinal tissue and feces of colorectal cancer patients, and the level of AI-2 produced by the intestinal flora correlates with tumor immunity. Ismail et al. (2016) revealed that an increase in AI-2 neutralizes dysbiosis in the mouse intestine and is associated with Firmicutes. Therefore, AI-2 plays an influential role in the synchronized behavior of intestinal flora. Furthermore, epithelial cells may activate QS by secreting a mimic of AI-2 in response to refactoring of the junction (Ismail et al., 2016). Thus, we hypothesized that the role of the probiotic complex in intestinal inflammation is associated with the AI-2/LuxS QS of LAB since various characteristics of the bacteria are regulated by AI-2, along with numerous other types of behaviors.

*Lactiplantibacillus plantarum* AB-1 and *L. plantarum* SS-128 are the two probiotics isolated from traditional dairy products and the fish intestine, respectively, and have been shown to produce AI-2 and exert beneficial effects, such as the production of antimicrobials (Li Q. et al., 2019; Qian et al., 2022). The present study investigated the ameliorative effect of the two wild-types and corresponding *luxS* mutant strains (AI-2-defective mutants) on mice with DSS-induced colitis to assess a more efficient treatment for human IBD.

## Materials and methods

### Materials

The primary antibody anti-mouse β-actin (R1011), lactic dehydrogenase (LDH) assay kit, Methylthiazolyldiphenyl-tetrazolium bromide (MTT), phenylmethanesul-fonyl fluoride, 3% H_2_O_2_ solution and Dextran Sulfate Sodium Salt (DSS) with the molecular weight ∼ 40,000 Da were purchased from Merck/Sigma-Aldrich Co., Ltd. (Shanghai, China). The Pierce Rapid Gold BCA assay kit, phosphate-buffered saline (PBS), and ELISA assay kits for mouse inflammatory cytokines, and the secondary antibodies of goat anti-mouse IgG H&L (HRP) (31,430) and goat anti-rabbit IgG H&L (HRP) (31,460) were provided by Thermo Fisher Scientific Co., Ltd. (Shanghai, China). The primary rabbit monoclonal anti-heat shock protein-25 (Hsp-25), anti-claudin-4, anti-occludin, and anti-nuclear factor kappa B (NF-κB) p65 were obtained from Affinity Biosciences (Changzhou, China).

### Bacterial growth and autoinducer-2 assay

Lactic acid bacteria was first propagated twice in Man Rogosa Sharpe broth (MRS; BD, Sparks, MD, United States) at 37°C for the bacterial growth assay for 36 h. During the incubation, the bacterial density of the samples was determined for the growth curve every 4 h and the cell-free supernatant was taken for the measurement of the AI-2 activity. The AI-2 activity was operated based on the modified approach (Bassler et al., 1997). The reported strain *Vibrio harveyi* strain BB170 was cultured in AB medium at 28°C for 16 h, then diluted 5,000 times. The tested sample was added to the diluted bacterial solution at a final concentration of 10% (vol/vol). The white, flat-bottomed, 96-well plates were shaken at 110 rpm at 28°C (PBS was used as the negative control). Light production was measured half-hourly by the SpectraMax i3x microplate reader (Molecular Devices, San Jose, CA, United States) in the bioluminescence mode. AI-2 activity was analyzed as the difference compared with the bioluminescence level in the control group and presented as relative luminescence units (RLUs).

### Preparation of bacteria for experiments

*Lactiplantibacillus plantarum* SS-128 was isolated from the intestine of *Sebastodes fuscescens* (Houttuyn) in Qingdao Province (China). *L. plantarum* AB-1 was a kind gift from Prof. Heping Zhang (Inner Mongolia Agricultural University, Hohhot, China) isolated from the horse milk wine. The *luxS* mutants (AI-2 deficient mutant) were constructed through two-step homologous recombination by the plasmid named pFED760. The pFED760 was a kind gift from Prof. Yiyong Luo (Jiangxi Normal University, Jiangxi, China).

### Caco-2 cell experiments

Fully differentiated Caco-2 cells model were used for assessing the physiological response of enterocytes to various oxidant stresses (Cilla et al., 2018). Caco-2 cells were kindly provided by Stem Cell Bank, Chinese Academy of Sciences (Shanghai, China). The cells were maintained in Dulbecco’s Modified Eagle’s Medium with 10% fetal bovine (Shanghai XP Biomed Ltd., Shanghai, China) at 37°C in 5% CO_2_ atmosphere.

Intracellular reactive oxygen species (ROS) were measured using the ROS Assay Kit (Beyotime Biotechnology, Shanghai, China). Cells were seeded in 48-well plates at the density of 2.0 × 10^4^ cells per well for 48 h. Then 10 μM DCFH-DA (Beyotime Biotechnology, Shanghai, China) in serum-free culture medium cells were added into the cells for 20 min, and after washing with the Dulbecco’s PBS, the DCF (Beyotime Biotechnology, Shanghai, China)-loaded cells were incubated with or without 3-fold diluted cell-free filtrate in DMEM (CFF) of LAB and treated with 1 mM H_2_O_2_ for 30 min (Qian et al., 2022). MRS broth was used as blank control, and exogenous 4,5-Hydroxybiphenyl-2,3-pentanedione (DPD) concentration was 80 μM, which could spontaneously transform into AI-2 (Winzer et al., 2003). DPD was synthesized and purified according to a previously reported method (De Keersmaecker et al., 2005). The fluorescence was detected at 488 nm excitation and 525 nm emission.

To assay LDH release, the cells were exposed to 1.0 mM H_2_O_2_, followed by incubation with or without CFF of LAB for 30 min before the media supernatant was harvested to measure the LDH with the assay kit as per the manufacturer’s instructions. The LDH release for the treatment cells was calculated as the percentage of absorbance in relation to the untreated cells. For apoptosis assay, cells were seeded in 96-well plates at a density of 5 × 10^3^ cells per well in 96-well plates and cultured for 72 h. Then the cells were exposed to 1.5 mM H_2_O_2_ with the incubation of with or without CFF of LAB for 60 min. After that, the cells were washed with Dulbecco’s PBS three times, followed by a 4 h incubation with 0.5 mg/mL MTT, and then were incubated with dimethyl sulfoxide to read at 570 nm. The cell viability was calculated as the percentage of absorbance in relation to the untreated cells.

### Animal experiments

#### Animals

Male 6-week-old C57BL/6 mice weighing 19.0–21.0 g were purchased from Jinan Pengyue Experimental Animal Breeding Co., LTD. (Jinan, Shandong, China). Mice were kept individually (one cage per mouse) in a room with 25°C and 55–65% relative humidity (12–12 h light-dark schedule) and given a pelletized LAD2001 standard diet (Trophic Animal Feed High-Tech Co., Ltd., Nantong, China) deionized water *ad libitum*. The animal scheme was agreed by the Committee on the Ethics of Animal Experiments of the Ocean University of China (no. SPXY20200910). The mice handlings were carried out ethically through the principles of the National Institutes of Health (NIH) *Guide for the Care and Use of Laboratory Animals*. In order to reduce suffering, all the experimental processes were completed under anesthesia.

#### Colitis induction protocol

After 5 days of adaptive growth, the mice were randomly divided into seven groups (six mice in each group, 42 mice in total) conducted simultaneously, which were the normal control group, DSS control group (PBS + DSS) and five LAB groups (*Lactobacillus rhamnosus* GG + DSS, AB-1 + DSS, Δ*luxS*/AB-1 + DSS, SS-128 + DSS and Δ*luxS*/SS-128 + DSS). As a reference strain, *L. rhamnosus* GG has been considered to ameliorate colitis in previous research (Choi, 2004; Son et al., 2019). The acute colitis was induced by DSS administration in the drinking water, according to the published method (Guo et al., 2020). The normal control group was given deionized water during the 7-day test period, the other groups were given 3% DSS (W/V) in deionized water, and all groups were given deionized water after 4 days (Figure 3A). The normal group and DSS control group were incubated with 200 μL PBS, and the LAB groups were given 200 μL PBS containing 1 × 10^9^ CFU/mL of five different LAB in the form of gavage. Mice were gavaged with LAB as a concomitant drug administration during DSS treatment and sacrificed on the seventh day.

#### Clinical evaluation of colitis

Food intake, body weight (BW), and symptoms of colitis were recorded every day. The Disease Activity Index (DAI) for ulcerative colitis is a total score of stool consistency, rectal bleeding, and BW loss (Supplementary Table 1). Serum of blood samples was obtained after the animals were euthanized (4,000 rpm for 15 min at 4°C). Every whole removed colon was measured in its natural state. After rinsing the colon thoroughly twice with PBS, it was cut into pieces within 0.5–1.0 cm for later use.

#### Histopathological analysis

The distal colon was first soaked in paraformaldehyde and then embedded in paraffin. The tissues were stained with hematoxylin and eosin (H&E) and alcian blue/periodic acid-Schiff (AB-PAS) for histopathology, respectively, followed sectioned to 5 μm thin. The Blind histological lesion score was described by a Nikon DS-Ri2 digital camera (Tokyo, Japan) (Zhao et al., 2020). The mucosal damage scoring system of Laroui et al. (2012) was employed to evaluate the degree of colitis.

#### Measurement of myeloperoxidase activity

The KZ-III-F TissueLyser (Servicebio, Wuhan, China) was used to homogenize the colon tissues (20 ± 5 mg) in ice-cold PBS containing 5% hexadecyltrimethylammonium bromide. After centrifugation at 13,000 g for 5 min (4°C), the supernatant of the sample (10 μL) was added to an active dianisidine substrate (200 μL, including 0.2 mg/mL o-dianisidine hydrochloride and 0.002% H_2_O_2_). The 96-well plate containing the mixture was placed on a microplate reader to measure the change in optical density at 450 nm. The myeloperoxidase (MPO) activity was expressed as units/g tissue.

#### Quantification of cytokine and protein expression in the colon

The colon (900 ± 10 mg) was homogenized in RIPA lysis buffer and phosphatase inhibitor cocktail with the KZ-III-F TissueLyser followed by centrifugation for 10 min at 12,000 g (4°C). The contents of tumor necrosis factor (TNF)-α, Interleukin (IL)-1β, and IL-6 in supernatants were determined by ELISA kit. The Hsp-25, claudin-4, occludin, and NF-κB in the tissue extract were quantified by western blotting analysis.

#### Quantitative real-time polymerase chain reaction

Total RNA was extracted by using Trizol reagent (1 mL), chloroform (0.2 mL), iso-propanol (0.5 mL), and 75% ethanol solution (1 mL), followed by reverse transcription into cDNA with the HiScript^®^ III RT SuperMix (Vazyme Biotech, China). qPCR was performed on a Bio-Rad CFX Opus 96 RT-PCR instrument (Hercules, CA, United States). The primer sequence is demonstrated in Table 1 with the *gapdh* used as reference genes. Repeat each experiment at least three times to analyze the data following the 2^–△△CT^ method (Zhao et al., 2020).

**TABLE 1:** Primer sequences used for qRT-PCR and PCR analysis.

Gene name	Forward primer (5′-3′)	Reverse primer (5′-3′)
*Gapdh*	TGGAGAAACCTGCCAAGTATGA	TGGAAGAATGGGAGTTGCTGT
*muc2*	GCTGACGAGTGGTTGGTGAATG	GATGAGGTGGCAGACAGGAGAC
*tff3*	CCGTGGTTGCTGTTTTGAC	GCCTGGACAGCTTCAAAATG
*zo-1*	GCTTTAGCGAACAGAAGGAGC	TTCATTTTTCCGAGACTTCACCA
16S RNA	ACTCCTACGGGAGGCAGCA	GGACTACHVGGGTWTCTAAT

#### Western blotting

The protein concentrations in cell lysates and tissue extracts were tested by Pierce™ Rapid Gold BCA protein assay kit. The denatured samples within the loading buffer were adsorbed to the PVDF membrane by SDS-PAGE for separation. β-actin was used as an internal control. Chemiluminescence was observed by an Immobilon^®^ ECL Ultra Western HRP Substrate (EMD Millipore, Burlington, MA, United States) on a ChemiDoc MP imager (Bio-Rad Laboratories, Inc., Hercules, CA, United States).

#### Analysis of gut microbiota by high-throughput sequencing

Seven fecal samples were chosen from each group and analyzed by Shanghai Majorbio Bio-Pharm Technology Co., Ltd. (Shanghai, China). The DNA was checked by agarose gel electrophoresis and quantified using a NanoDrop ND-2000 spectrophotometer (Thermo Scientific, Wilmington, NC, United States). The V3-V4 regions of the 16S rDNA were amplified with forwarding and reverse primer and sequenced (Table 1). The sequencing errors and chimeras were removed according to previous procedures (Schloss et al., 2011; Kozich et al., 2013). Low-quality reads and undesirable sequences were removed, and chimera sequences were screened by UCHIME (Edgar and Flyvbjerg, 2015). Operational taxonomic units (OTU) were obtained by sequence alignment and division. α-diversity was assessed by phylogenetic information Chao and Principal Coordinate analysis (PCoA) (Quast et al., 2012). QIIME software (v1.9.1) was used to make a histogram of species in the phylum, family, and genus classification levels to analyze the different species proportions in each sample.

#### Fecal short-chain fatty acids analysis

Short-chain fatty acids (SCFs) from stool samples were extracted in an aqueous solution and further analyzed on gas chromatography according to the method previously reported by Zhao et al. (2020). Analytes were quantified in a series of standard stock solutions.

### Statistical analysis

Data were presented as means ± standard deviations. One-way analysis of variance (ANOVA) with Duncan’s *post hoc* test was used to analyze the mean differences on SPSS software version 24.0 (SPSS, Inc., Chicago, United States). The probability level *P* < 0.05 indicates a significant difference.

## Results

### Changes in the growth and autoinducer-2 production

**FIGURE 1:**
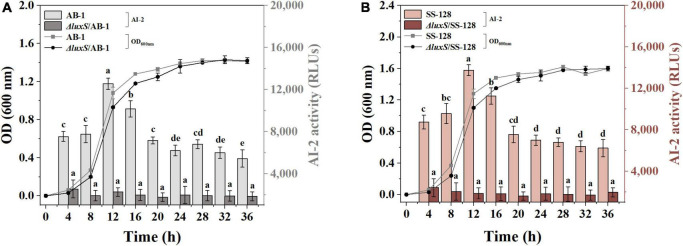
AI-2 activity and cell density of **(A)** AB-1 and Δ*luxS*/AB-1 and **(B)** SS-128 and Δ*luxS*/SS-128. The data are expressed as the mean ± standard deviation (*n* = 3), with different letters (a–e) corresponding to statistically significant differences (*P* < 0.05).

To demonstrate AI-2 secretion deficiency in the *luxS* mutants of *L. plantarum* SS-128 and *L. plantarum* AB-1 (*L. plantarum*Δ*luxS*/SS-128 and *L. plantarum*Δ*luxS*/AB-1, respectively), the AI-2 activity in the supernatants of the wild type and *luxS* mutant strains was monitored by bioluminescence. Knockout of *luxS*, which encodes *S*-ribosylhomocysteine lyase involved in methionine metabolism, ultimately leads to the loss of AI-2 (Pereira et al., 2013). The growth behavior of *L. plantarum*Δ*luxS*/SS-128 and *L. plantarum* Δ*luxS*/AB-1 was detected at 37°C. As shown in Figure 1, an increase in the somatic cell density of AI-2-deficient mutants was slower than that of the corresponding wild-type strains. Notably, the accumulated concentration of AI-2 peaked in the late exponential phase and was slowly decreased during the stationary phase. These results are supported by the data of Kaur et al. (2020), who reported that the accumulation of AI-2 continues until the late exponential phase. The output of AI-2 was maximal at a high reproduction rate; hence, LAB cultured for 12 h were prepared for subsequent experiments.

### Beneficial effects of lactic acid bacteria metabolites on the intestinal barrier in Caco-2 cells

**FIGURE 2:**
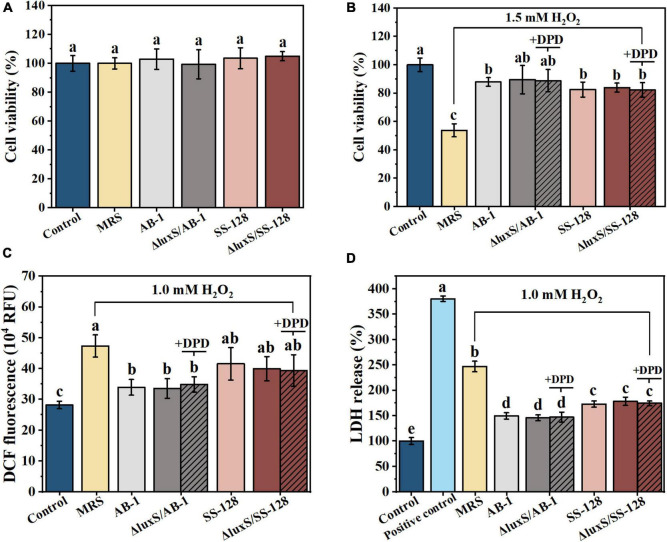
Beneficial effects of cell-free filtrate of LAB on H_2_O_2_-treated Caco-2 cells after incubation for 12 h: **(A)** MTT assays; **(B)** cell viability; **(C)** intracellular ROS; and **(D)** LDH release. The data are expressed as the mean ± standard deviation (*n* = 3), with different letters (a–d) corresponding to statistically significant differences (*P* < 0.05). ROS, reactive oxygen species; LDH, lactate dehydrogenase.

The injury of epithelial cells directly contributes to the disruption of the enteric epithelial barrier and systemic inflammatory complications (Guo et al., 2022). Figure 2A shows the lack of significant cytotoxicity toward Caco-2 cells upon incubation with CFF of the tested LAB strains according to the data of an MTT assay (*P* > 0.05). The effect of the signal-ling molecules of LAB on epithelial cells was further tested in CFF of AI-2-deficient mutants based on exogenous DPD. The amount of DPD was determined as a concentration of AI-2 secreted by the wild-type strains during 12 h.

The data of Figure 2B indicated that the treatment with 1.5 mM H_2_O_2_ for 60 min resulted in an almost 50% loss of viability of Caco-2 cells. The damage was significantly alleviated by incubation with CFF of LAB (*P* < 0.05). However, exogenous AI-2 did not significantly change the degree of viability rescue (*P* > 0.05). These results suggested that the protective effects of CFF of the wild-type strains were comparable to those of the *luxS* mutants in the context of oxidation-induced apoptosis of intestinal epithelial cells, and this effect was independent from AI-2.

To explore the protective effect induced by the antioxidant characteristics of AI-2 produced by LAB on oxidative stress-mediated mucosal damage, DCF fluorescence was measured in Caco-2 cells (Figure 2C). Treatment with 1 mM H_2_O_2_ for 0.5 h increased the fluorescence signal of the cells, and this increase was significantly inhibited by incubation with CFF (*P* < 0.05). These results indicated that CFFs of the wild-type and AI-2-deficient mutant strains were equally effective in attenuating H_2_O_2_-induced intracellular ROS outbursts. ROS damage resulted in subsequent significant leakage of cytoplasmic LDH after the treatment with 1 mM H_2_O_2_ for 0.5 h (Figure 2D). This phenomenon was also partially reversed by the treatment with CFF, and CFF of Δ*luxS*/AB-1 showed a considerably better inhibitory effect on LDH leakage than that of Δ*luxS*/SS-128. These results suggested an equivalent cytoprotective effect of CFFs of the wild-type and AI-2-deficient mutant strains against ROS-induced enterocyte injury.

Based on the data on cell viability, DCF fluorescence and LDH release, CFF of LAB was equally effective in preventing damage to the gut epithelial barrier, while exogenous AI-2 did not influence these protective effects.

### Changes in body weight, food intake, and disease activity index

**FIGURE 3:**
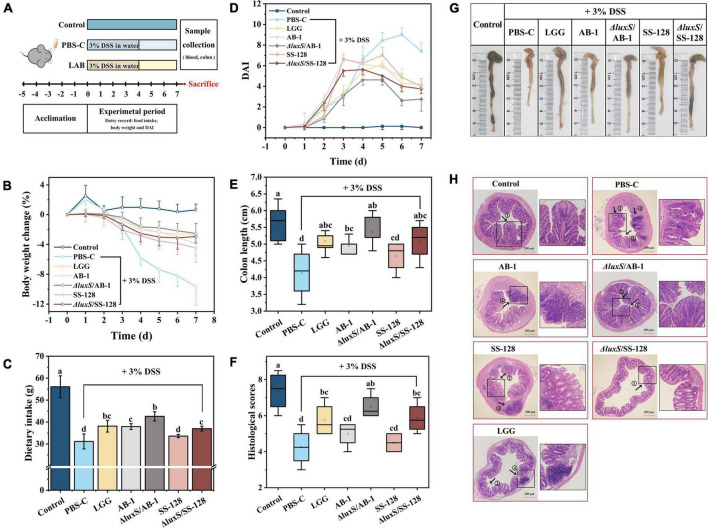
Effects of orally administered LAB on clinical and histological symptoms of DSS-induced colitis in mice: **(A)** experimental design; **(B)** body weight changes; **(C)** dietary intake; **(D)** DAI; **(E)** colon length; **(F)** histological scores; **(G)** typical images of the colon length measurements, and **(H)** representative images of H&E-stained colonic sections. The data are expressed as the mean ± standard deviation (*n* = 6). Different letters (a–d) correspond to statistically significant differences (*P* < 0.05). Histopathological images of the colon tissues: arrow 1 indicates the intact crypts; arrow 2 indicates colonic mucosal erosion; arrow 3 indicates a severe reduction in goblet cells; and arrow 4 indicates inflammatory cell infiltration, with preservation of the intact surface epithelium. DAI, disease activity index.

To assess the ameliorative effect of LAB on DSS-induced colitis in mice, the BW, food intake and DAI of the animals were recorded and are shown in Figure 3. The BW in the DSS control group was significantly decreased from day 3 compared to that in the normal control group (*P* < 0.05) (Figure 3B). However, a notably lower BW loss was observed in the LAB treatment groups (*L. rhamnosus* GG, AB-1, Δ*luxS*/AB-1, SS-128, and Δ*luxS*/SS-128) starting from day 4 (*P* < 0.05). The total food intake of mice in the DSS control group (31.2 ± 3.4 g) was the lowest among all groups (Figure 3C) during the 7-day experimental period (*P* < 0.05). Mice treated with Δ*luxS*/AB-1 manifested an increase in the total food intake (42.6 ± 2.1 g) compared with other treatment groups (*P* < 0.05). All groups treated with DSS manifested an increase in the values of DAI, indicating considerable BW loss, diarrhea and bloody stool starting from day 2 (Figure 3D). Consistent with observations of the BW changes, oral administration of LAB alleviated DAI starting from day 6 (*P* < 0.05), especially in the case of the AI-2-deficient mutants, indicating an enhanced ameliorating effect on the symptoms of colitis in mice.

### Evaluation of the colon length and mucosal integrity

A considerable reduction in the colon length was identified as the biological marker of colitis severity in mice (Figures 3E,G). Mice treated with Δ*luxS*/AB-1 and Δ*luxS*/SS-128 had the colon lengths of 5.4 ± 0.3 and 5.1 ± 0.6 cm, respectively, which were not significantly different from that in the normal control group (*P* > 0.05). The H&E-stained sections shown in Figure 3H and the histological scores shown in Figure 3G revealed intact mucosa without inflammatory infiltration in normal control mice. After DSS administration, mice manifested notable inflammatory histopathological signs, such as crypt distortion, caliciform cell loss in the mucosa and submucosa and inflammatory cell infiltration in the lamina propria. However, the histological scores of mice administered LAB were significantly lower than those in the DSS control group (*P* < 0.05). The treatment with AI-2-deficient mutants alleviated colon injury to a greater extent than the effect induced by the wild-type strains. Overall, the data implied that supplementation with LAB ameliorated DSS-induced colonic injury in mice. Comparison with the wild-type strains indicated that the AI-2-deficient mutants weakened the symptoms of colitis in mice more effectively.

### Myeloperoxidase activity and inflammatory cytokine expression

**FIGURE 4:**
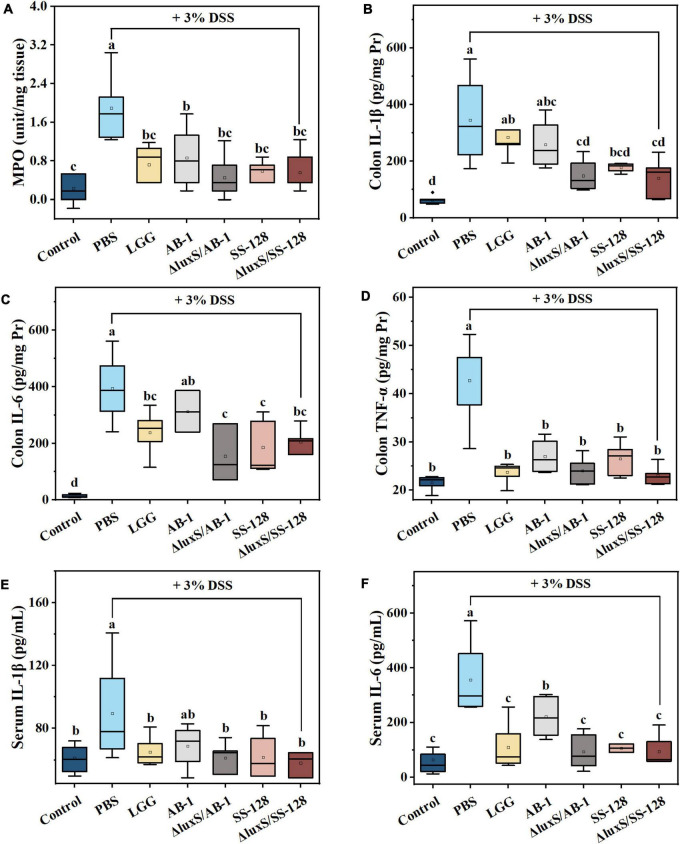
Effects of LAB supplementation on the inflammatory responses in mice with DSS-induced colitis. The colonic levels of **(A)** MPO activity, **(B)** IL-1β, **(C)** IL-6, and **(D)** TNF-α; the serum levels of **(E)** IL-1β, and **(F)** IL-6. The data are ex-pressed as the mean ± standard deviation (*n* = 6). Different letters (a–d) correspond to statistically significant differences (*P* < 0.05).

Myeloperoxidase activity is positively correlated with the severity of IBD and is released by neutrophils (Wagner et al., 2008; Ahmad et al., 2020). As illustrated in Figure 4A, LAB treatment significantly inhibited an increase in MPO activity caused by DSS and restored the MPO levels to the values that were not significantly different from that in the normal control group (*P* > 0.05).

**FIGURE 5:**
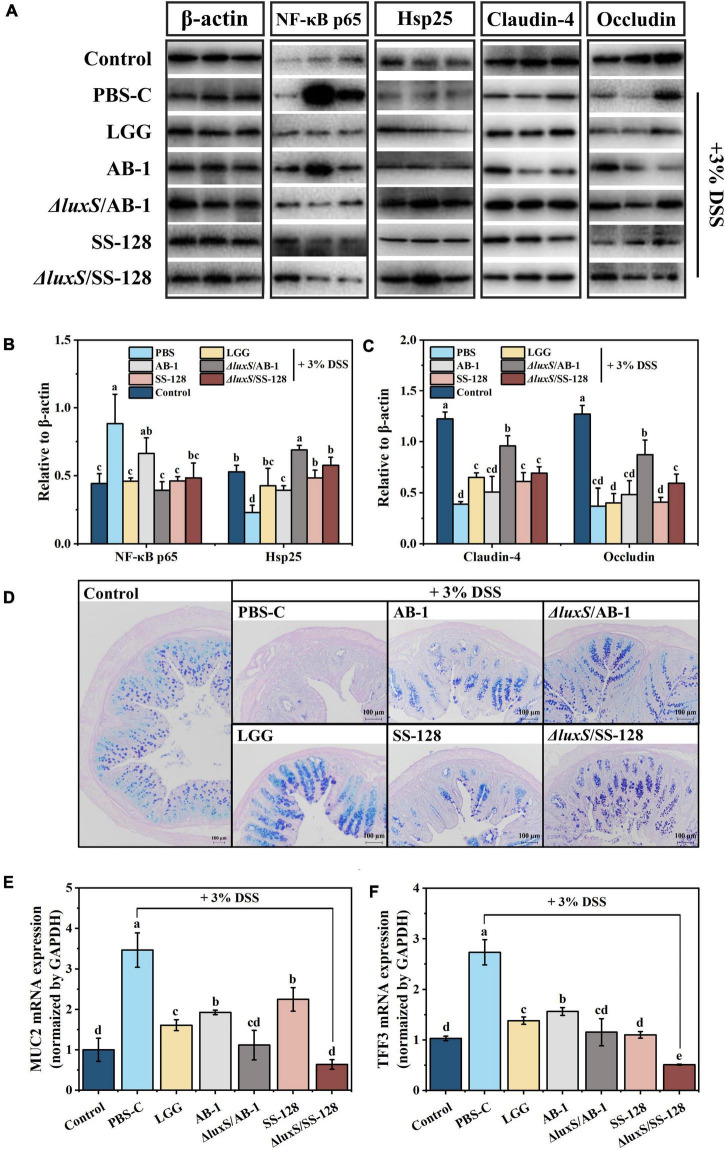
Effects of LAB supplementation on the gut barrier integrity in mice with DSS-induced colitis: **(A)** imaging and **(B,C)** densitometric analysis of western blots; **(D)** typical images of AB-PAS staining; mRNA expression of **(E)** MUC2 and **(F)** TFF3. The values are shown as the mean ± standard deviation of a representative experiment (*n* = 6). Different letters (a–d) correspond to statistically significant differences (*P* < 0.05).

During the pathogenesis of DSS-induced colitis, we investigated the expression of the proinflammatory cytokines IL-6, IL-1β, and TNF-α, which are abundantly recruited in mice during DSS-induced colitis (Lee et al., 2011; Soufli et al., 2016). The data of Figures 4B–D indicated that DSS induced an approximately 2–10-fold increase in the colonic levels of the cytokines compared to the normal control group. This phenomenon was markedly suppressed by administration of LAB, and the inhibitory effect of the AI-2-deficient mutants was better than that in the *L. rhamnosus* GG-treated group. Thus, the AI-2-deficient mutants prevented the initiation of the inflammatory response more efficiently than the wild-type strains. We also demonstrated that elevation of peripheral cytokines (IL-6 and IL-1β) was significantly suppressed by the administration of LAB (*P* < 0.05) (Figures 4E,F). Moreover, the results of western blotting shown in Figures 5A,B indicated that oral administration of AI-2-deficient mutants notably decreased the expression of NF-κB, which is the crucial regulator of the signal transduction of inflammatory cytokines assayed as described above. The results of the assays of MPO activity and inflammatory cytokines indicated that the AI-2-deficient mutants had equivalent anti-inflammatory potential that was superior to that of the corresponding wild-type strains.

### The diversity of the gut microbiota

**FIGURE 6:**
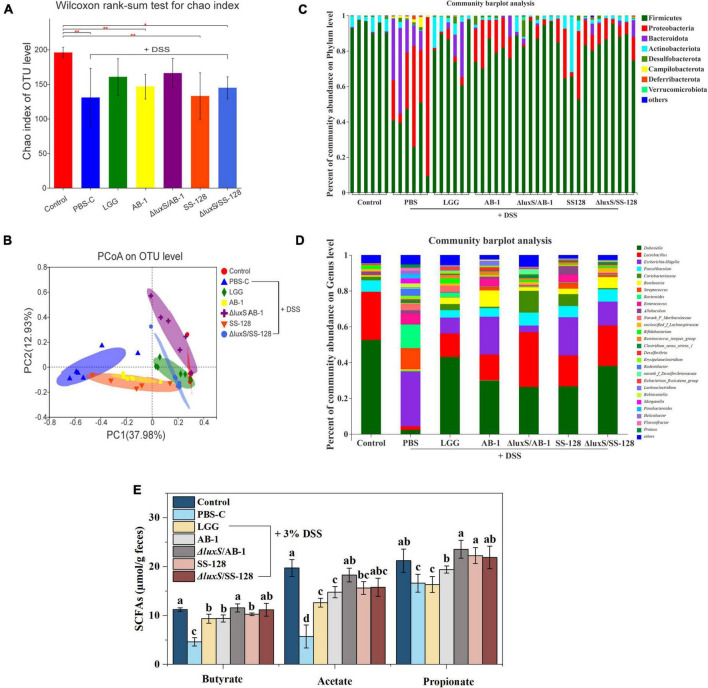
Analysis of bacterial diversity and SCFAs in the groups of mice after DSS administration: **(A)** Chao index; **(B)** PCoA of the beta diversity of intestinal flora in mice **(C)** at the phylum level and **(D)** at the genus level; **(E)** the fecal levels of short-chain fatty acids. The data are expressed as the mean and standard deviation (*n* = 6). Different letters (a–d) correspond to statistically significant differences (*P* < 0.05).

A total of 2,521,367 high-quality 16S rRNA sequences were obtained from 42 samples from seven experimental groups. A total of 393 operational taxonomic units (OTUs) were obtained at 97% identity after reaching the required sequencing depth and equal clustering of each sample, and the number of OTUs ranged from 202 to 269 per sample. The Chao index, which estimates the number of OTUs, indicated that the microbial diversity in DSS-treated mice was significantly lower than that in normal control mice (*P* < 0.05) (Figure 6A).

The alterations in intestinal flora were visualized based on the number of unique and common OTUs in the groups. Supplementary Figure 1A shows 104 OTUs overlapping in the Venn diagram of the seven groups and 115 OTUs overlapping within the five groups (Supplementary Figure 1B), further indicating that intestinal microbial diversity was destroyed in DSS-treated mice. However, LAB treatment, especially the treatment with the AI-2-deficient mutants, notably resulted in the preservation of microbial diversity. The data of PCoA indicated the lack of an overlap between the normal control and DSS control groups, and the treatment with AI-2-deficient mutants successfully altered the microbiome from that detected in the DSS group (Figure 6B). These results suggested that the AI-2-deficient mutants are superior to the wild-type strains in ameliorating the changes in the intestinal microbial community.

### Microbiota at the phylum, family, and genus levels

Figure 6C shows the relative abundance of the microbes in individual mice at the phylum level, which was dominated by Firmicutes, Actinobacteria, and Bacteroidetes. The relative abundance of Proteobacteria and Bacteroidetes in mice treated with DSS was significantly increased (*P* < 0.05), and the abundance of Firmicutes was decreased. In contrast, the administration of LAB changed the proportions of the microbiome composition at the phylum level, especially in the case of the AI-2-deficient mutants.

At the family level (Supplementary Figure 1C), the relative abundance of Enterobacteriaceae, Streptococcaceae, Bacteroidaceae, and Enterococcaceae was significantly higher in the DSS treatment group than that in the normal control group, and the abundance of Erysipelotrichaceae, Lactobacillaceae, and Bifidobacteriaceae was decreased. However, the treatment with the AI-2-deficient mutants upregulated the abundance of Atopobiaceae and Bifidobacteriaceae and downregulated the abundance of Enterococcaceae, Tannerellaceae, and Helicobacteraceae compared with those in the DSS treatment group. At the genus level, LAB administration induced a recovery of intestinal flora dominated by *Dubosiella*, *Lactobacillus*, and *Faecalibaculum* (Figure 6D). In particular, the AI-2-deficient mutants significantly reduced the abundance of *Escherichia-Shigella* and *Desulfovibrio* and increased the abundance of *Lactobacillus* and *Faecalibaculum*.

### Analysis of short-chain fatty acids

Short-chain fatty acids are generated during fiber fermentation by Firmicutes and Bacteroides and have anti-colitis effects (Thompson et al., 2015; Bian et al., 2020). Three major SCFAs, including acetate, propionate and butyrate, were detected, and the concentrations of these acids were markedly decreased by DSS treatment (*P* < 0.05) (Figure 6E). However, LAB administration considerably enhanced the SCFA levels (*P* < 0.05); in particular, there were no significant differences between the group treated with Δ*luxS*/AB-1 and the normal control group.

### Analysis of intestinal barrier function

Heat shock proteins (Hsp) can be induced by specific stress factors, and Hsp up-regulation protects intestinal epithelial cells from oxidative damage (Bian et al., 2020). The data of Figures 5A,B illustrate differential expression of Hsp, suggesting that oral administration of the AI-2 deficient mutants enhanced the mobilization of intestinal protective function in mice with enteritis. The tight junction proteins located between the cells in the lumen wall effectively block the invasion of bacteria and toxins, preventing the activation of the abnormal immune responses (Ichikawa-Tomikawa et al., 2011; Capaldo et al., 2017). Therefore, occludin, zonula occludins (ZO), and claudin family proteins, which are essential components of the tight junctions, were investigated at both the transcript and protein levels. Comparison with the normal control group indicated that the DSS control group showed a reduction in the levels of claudin-4 and occludin within the colon of mice with colitis, and oral treatment with LAB partially reversed this decrease. Notably, the treatment with the AI-2-deficient mutants significantly inhibited a DSS-induced decrease in claudin-4 and occludin (*P* < 0.05) (Figures 5A,C). The data of RT–qPCR indicated that supplementation with LAB reversed a decrease in the expression of ZO-1 compared with that in the DSS control group (Supplementary Figure 2).

The mucus layer produced by intestinal goblet cells is the primary defense that limits the exposure of epithelial cells to various harmful substances (Mashimo et al., 1996). Previous studies have proven that the barrier function of the mucous layer is associated with the initiation of IBD and deterioration induced by IBD (Ijssennagger et al., 2016). As shown in Figure 5D, the results of AB-PAS staining indicated that a decrease in the number of goblet cells in DSS-induced mice was significantly lower than that in the normal control group; in particular, LAB supplementation reversed the changes in these values to the normal level. The gel-forming mucins are produced and secreted by intestinal goblet cells, predominantly including mucin 2 (MUC2), and trefoil factor family peptide 3 (TFF3), which are the bioactive peptide derivatives that participate in the maintenance of epithelial cells; these mucins are considered indicators of goblet cell protection (Ijssennagger et al., 2016). Some studies reported that the accumulation of MUC2 and TFF3 in IBD is increased, implying a colonic stress response that induces certain changes in the mucus (Mashimo et al., 1996; Cheng et al., 2020). Similarly, the intervention using the AI-2-deficient mutants significantly reduced DSS-induced expression of MUC-2 and TFF3 (*P* < 0.05) (Figures 5E,F).

The results of the assays of intestinal barrier indicators indicated that the AI-2-deficient mutants were able to more efficiently ameliorate colitis in mice by reducing the intestinal inflammatory response and breakdown of the intestinal barrier.

### Analysis of correlations between the microbiome and other biochemical indices

**FIGURE 7:**
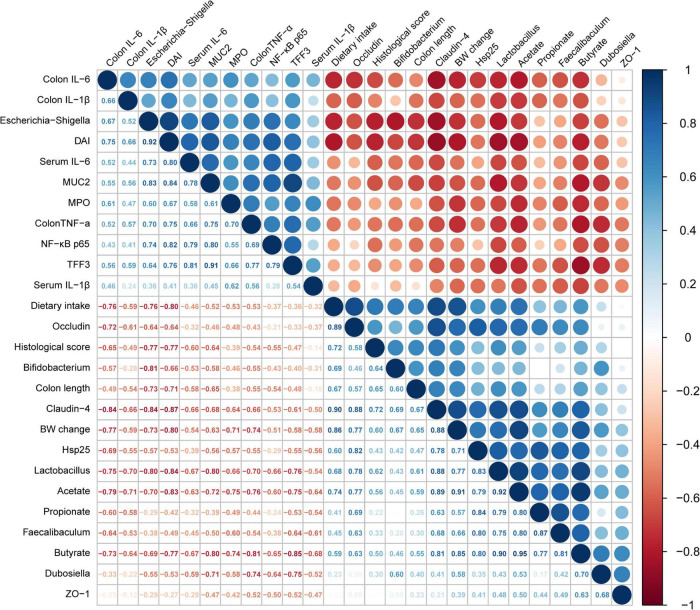
Analysis of the correlations between the relative abundance of intestinal flora and biochemical indices. The size and color of the circles indicate the corresponding index levels and values, with red representing a negative value and blue representing a positive value.

To further verify the correlations between the changes in intestinal flora induced by AI-2 from LAB and the inflammatory response of mice with colitis, Spearman correlation analysis was used to obtain the results (Figure 7). The data revealed significant correlations between enteric microorganisms and the indicators of inflammation (*P* < 0.05). For instance, *Lactobacillus*, which was enriched by LAB treatment, was significantly positively correlated with claudin-4 (*r* = 0.88), Hsp25 (*r* = 0.83), and occludin (*r* = 0.78). However, *Lactobacillus* was negatively correlated with DAI (*r* = –0.67), colonic IL-6 (*r* = –0.75), and NF-κB p65 (*r* = –0.66). Similar results were detected in the gut microbiota using *Faecalibaculum*. As shown in Figure 7, the tight junction proteins (occludin and claudin-4), which are associated with the intestinal barrier, were positively correlated with potentially beneficial bacteria (*Lactobacillus* and *Bifidobacterium*) and SCFS (acetate and butyrate) and negatively correlated with intestinal inflammatory factors (IL-6 and TNF-α) and potentially harmful bacteria (*Escherichia*-*Shigella*). The results showed that beneficial effects of various LAB strains in mice with colitis exhibited significant regularity. Thus, AI-2 produced by LAB was closely associated with intestinal flora of mice with colitis, thus influencing the integrity of the intestinal barrier and other parameters.

## Discussion

Probiotic LAB have been considered human health promoters due to their unique advantages, including fermentation, acid and bile tolerance, colonization in the intestinal mucosa and residence in the gastrointestinal tract (Wang et al., 2020; Tong et al., 2021). The data of the literature confirm clinically proven health effects of LAB, such as diarrhea prevention and lactose intolerance relief, and most of these effects are considered to be related to probiotics that regulate the gut microbiome (Doron and Gorbach, 2006; Papanicolas et al., 2020). In addition, cumulative evidence indicates the substantial effect of LAB in alleviating IBD (Son et al., 2019; Sun et al., 2020). The present study investigated the effect of AI-2/LuxS-mediated QS of *L. plantarum* on DSS-induced acute colitis in mice and the ability of these LAB to alleviate gut dysbiosis.

**FIGURE 8:**
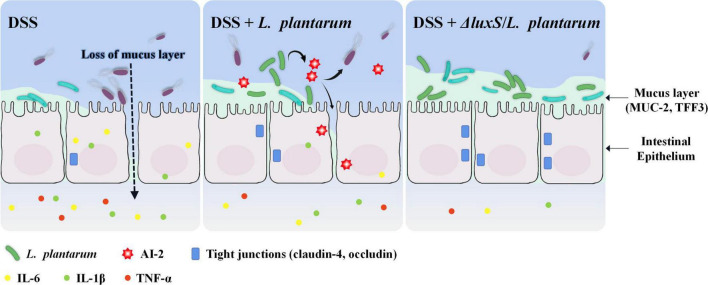
Schematic presentation of the anti-colitis effect of AI-2-deficient *Lactiplantibacillus plantarum*.

Oral administration of DSS in mice induces epithelial injury and compromises barrier integrity, subsequently exposing the mucosa to the luminal antigens and triggering a series of violent inflammatory immune responses (Wirtz et al., 2017). The data of the present study included the changes in BW, dietary intake, DAI and colonic indicators to indicate that the AI-2-deficient mutants ameliorated DSS-induced colitis in mice, and the administration of the corresponding wild-type strains did not elicit some of these effects. DSS-induced infiltration of neutrophils and macrophages into mouse colonic mucosa induced the secretion of considerable amounts of inflammatory cytokines and MPO, and the treatment with *L. plantarum* alleviated this aggravated inflammatory status. The results of the present study were consistent with the reported effects of LAB, including a reduction in the levels of proinflammatory mediators (Ahn et al., 2014; Wirtz et al., 2017). IL-6, IL-1β and TNF-α are essential proinflammatory mediators that accumulate in large quantities within the colon tissues with IBD symptoms, and NF-κB is a crucial regulator of these intracellular signaling pathways. As expected, the AI-2-deficient mutants were able to decrease the expression of NF-κB, thereby inhibiting the expression of the downstream inflammatory cytokines, such as IL-6 and TNF-α. This result was consistent with a study that demonstrated that anti-inflammatory activity of the lysate of *L. plantarum* K8 was mediated by inhibition of NF-κB activation (Ahn et al., 2014). The results of the present study showed that the ameliorating effect of the AI-2-deficient mutants on the symptoms of colitis was more pronounced than that of the wild-type strains (Figure 8).

Disorders of intestinal flora are one of the crucial factors in the pathogenesis of IBD, leading to intestinal mucosa stimulation and further mucosal inflammation. At the same time, intestinal inflammation alters the intestinal environment, resulting in the changes in the composition of intestinal flora (Wine et al., 2010; Matsuoka and Kanai, 2015). The results of correlation analysis of the microbiome and other biochemical indices obtained in the present study were similar to observations reported by other authors (Figure 7). The data of the present study on ROS detection, LDH release and cell viability indicated that CFF of the wild-type strains and corresponding AI-2-deficient mutants was equally effective in preventing damage to the gut epithelial barrier, and exogenous AI-2 did not influence these protective effects. Therefore, analysis of the present study focused on synchronous communications of intestinal microorganisms to demonstrate whether LAB-produced AI-2 has a specific impact on the restoration of the microbiota composition and amelioration of colitis.

Previous studies demonstrated that the diversity and abundance of the intestinal microbiome in DSS-treated mice are significantly different from those in healthy mice (Son et al., 2019). In the present study, oral administration of *L. plantarum* induced a specific recovery of intestinal flora, especially in the case of the AI-2-deficient mutants. At the phylum level, Proteobacteria were less abundant in the normal control group and were enriched in the mouse intestine in the DSS-treated group, and the proportion of Proteobacteria was significantly reduced in the *luxS* mutant-treated group. The *luxS* mutants effectively maintained the abundance of Actinobacteria. At the family level, *L. plantarum* treatment restored a decrease in the abundance of Lactobacillaceae caused by DSS treatment. Furthermore, the AI-2-deficient mutants reversed the DSS effect by reducing the percentage of Lachnospiraceae and Atopobiaceae and restoring the percentage of the Lactobacillaceae family. Lactobacillaceae and Bifidobacteriaceae are the most common probiotics, which can produce lactic, acetic and propionic acids and restore mucus growth, thus reducing intestinal pH and improving the intestinal environment (Doron and Gorbach, 2006; Schroeder et al., 2018). In combination with the SCFA determination, these results suggested that the AI-2 deficient mutants enhanced the abundance of intestinal probiotics to elevate the level of SCFAs, thus ameliorating intestinal inflammation in DSS-induced colitis in mice, and this effect of the mutants was more pronounced than the effects of the wild-type strains.

Intestinal epithelial cells and mucus layer effectively block destructive stimulatory substances from the underlying tissues, while the effects of DSS facilitate the passage of pathogenic microorganisms through the intestinal mucosal barrier, inducing damage and triggering infection (Martens et al., 2018). *Escherichia*-*Shigella* were enriched in mice administered DSS, suggesting that the damaged gut could have facilitated the overgrowth of these bacteria to increase inflammation. Consistent with the results of the present study, *Escherichia*-*Shigella* have been reported to enhance the permeability of the intestinal tract and aggravate colon inflammation in IBD patients (Sokol et al., 2006; Bian et al., 2020). Motility and adherence of these bacteria facilitate pathogen colonization (Schroeder et al., 2018). However, oral administration of Δ*luxS*/AB-1 reduced the abundance of *Escherichia*-*Shigella* to less than 5%, although all LAB inhibited the growth of these bacteria. Intestinal-related bacteria, such as *Escherichia coli* and *Salmonella typhimurium*, respond to AI-2 by activating the transcription of the *lsr* operon (Taga et al., 2003; Xavier et al., 2007; Ranava et al., 2021). AI-2 can be sensed by enterohemorrhagic *E. coli* (EHEC) to increase its swimming activity and is believed to be an essential sign of human enterohemorrhagic EHEC infection (Bansal et al., 2010).

In previous study, the number of sulfate-reducing bacteria (SRB) in the mucosal flora and feces of IBD patients was higher than that in the normal control group, and most SRB are the members of the genus *Desulfovibrio* (Ijssennagger et al., 2016). Sulfide produced by SRB reduces disulfide bonds in the mucus network, and the resulting breaks in the mucus barrier expose the epithelium to bacteria and toxins, causing inflammation. However, sensing of AI-2 by SRB induces an intercellular structure that allows metabolic exchange and energetic coupling between the bacteria (Ijssennagger et al., 2016; Tong et al., 2021). Therefore, comparison with the AI-2-deficient mutants indicated that AI-2 produced by the wild-type strains may stimulate the reproduction of harmful microorganisms and expression of virulence factors in the intestinal microbiota.

Wrzosek et al. (2013) demonstrated that *Faecalibacterium* is closely associated with the protection of the intestinal barrier by augmenting goblet cell differentiation and inducing the expression of the genes related to mucin glycosylation. The data of the present study indicated that *Faecalibacterium* was significantly enriched in AI-2-deficient mutant-treated mice and was positively correlated with an increase in butyrate (Figure 6D). Butyrate produced by *Faecalibacterium prausnitzii* induces the synthesis and production of mucin and improves the intestinal barrier function (Carlsson et al., 2013; Wrzosek et al., 2013). These considerations suggest that the AI-2-deficient mutants used in the present study synchronously regulated intestinal flora at the population level and thus may be able to improve the intestinal function by effectively alleviating the damage to goblet cells and restoring the levels of MUC2 and TFF3 to the normal values. In general, AI-2-deficient *L. plantarum* showed a better effect in maintaining gut health by regulating the quorum behavior of gut microbiota, thus protecting the intestinal barrier and inhibiting colonic and systemic inflammatory responses (Figure 8).

The results of the experiments using Caco-2 cells showed that exogenous AI-2 had no significant effect on the repair of injured epithelial cells (Figure 2). We hypothesized that when the intestinal mucus and surface cells are destroyed, AI-2 produced by LAB stimulates the remodeling of intestinal flora to increase susceptibility of intestinal cells to DSS colitis, which is not conducive to the repair of the intestinal epithelium. The AI-2 molecules produced by various members of the gut microbiome are considered to be differentially integrated into the virulence regulatory cascade (Hsiao and Zhu, 2020). Ismail et al. (2016) suggested that transboundary communications between eukaryotic cells and bacteria may be performed through the AI-2/LuxS QS system, although eukaryotic cells lack the *luxS* gene encoding AI-2 synthase. Therefore, the stimulating effect of AI-2 on injured intestinal epithelial cells requires additional exploration. The present study showed that AI-2 produced by LAB in the intestinal tract has a significant impact on the regulation of intestinal flora and stimulation of intestinal cells.

## Conclusion

Overall, the results of the present study demonstrated that AI-2-deficient *L. plantarum* was more efficient in maintaining intestinal health by regulating intestinal flora, thus protecting the intestinal barrier and inhibiting colonic and systemic inflammatory responses better than the wild-type strain. In addition, the negative effects of AI-2 on structurally impaired intestine need to be elucidated in the future to optimize the potential beneficial role of probiotics in the treatment of IBD.

## Data availability statement

The data presented in this study are deposited in the NCBI repository, accession number: PRJNA872119.

## Ethics statement

The animal study was reviewed and approved by the Committee on the Ethics of Animal Experiments of the Ocean University of China.

## Author contributions

YQ: data curation, writing—original draft preparation, and conceptualization. LM: methodology, software, and visualization. MZ: formal analysis and visualization. ZL: visualization, investigation, and funding acquisition. All authors contributed to the article and approved the submitted version.

## Abbreviations

AB-PAS: alcian blue/periodic acid-Schiff
AI-2: autoinducer-2
BW: body weight
CFF: cell-free filtrate
DAI: disease activity index
DSS: dextran sodium sulfate
IBD: inflammatory bowel diseases
IL: interleukin
H&E: hematoxylin and eosin
LDH: lactic dehydrogenase
MPO: myeloperoxidase
MUC2: mucin 2
NF- κ B: nuclear factor kappa B
QS: quorum sensing
ROS: reactive oxygen species
SCFs: short-chain fatty acids
SRB: sulfate-reducing bacteria
TFF3: trefoil factor family peptide 3
TNF: tumor necrosis factor.

## References

[B1] Ahmad G. Chami B. Liu Y. Schroder A. L. San Gabriel P. T. Gao A. (2020). The synthetic myeloperoxidase inhibitor AZD3241 ameliorates dextran sodium sulfate stimulated experimental colitis. *Front. Pharmacol.* 11:556020. 10.3389/fphar.2020.556020 33041796 PMC7522858

[B2] Ahn Y. S. Park M. Y. Shin J. H. Kim J. Y. Kwon O. (2014). Lysate of probiotic *Lactobacillus plantarum* K8 modulate the mucosal inflammatory system in dextran sulfate sodium-induced colitic rats. *Korean J. Food Sci. Anim. Resour.* 34 829–835. 10.5851/kosfa.2014.34.6.829 26761681 PMC4662199

[B3] Bansal T. Alaniz R. C. Wood T. K. Jayaraman A. (2010). The bacterial signal indole increases epithelial-cell tight-junction resistance and attenuates indicators of inflammation. *Proc. Natl. Acad. Sci. U.S.A.* 107 228–233. 10.1073/pnas.0906112107 19966295 PMC2806735

[B4] Bassler B. L. Greenberg E. P. Stevens A. M. (1997). Cross-species induction of luminescence in the quorum-sensing bacterium *Vibrio harveyi*. *J. Bacteriol.* 179 4043–4045. 10.1128/jb.179.12.4043-4045.1997 9190823 PMC179216

[B5] Bian X. Yang L. Wu W. Lv L. Jiang X. Wang Q. (2020). *Pediococcus pentosaceus* LI05 alleviates DSS-induced colitis by modulating immunological profiles, the gut microbiota and short-chain fatty acid levels in a mouse model. *Microb. Biotechnol.* 13 1228–1244. 10.1111/1751-7915.13583 32363766 PMC7264873

[B6] Capaldo C. T. Powell D. N. Kalman D. (2017). Layered defense: how mucus and tight junctions seal the intestinal barrier. *J. Mol. Med.* 95 927–934. 10.1007/s00109-017-1557-x 28707083 PMC5548832

[B7] Carlsson A. H. Yakymenko O. Olivier I. Håkansson F. Postma E. Keita ÅV. (2013). *Faecalibacterium prausnitzii* supernatant improves intestinal barrier function in mice DSS colitis. *Scand. J. Gastroenterol.* 48 1136–1144. 10.3109/00365521.2013.828773 23971882

[B8] Celiberto L. S. Bedani R. Rossi E. A. Cavallini D. C. U. (2017). Probiotics: the scientific evidence in the context of inflammatory bowel disease. *Crit. Rev. Food Sci. Nutr.* 57 1759–1768. 10.1080/10408398.2014.941457 25996176

[B9] Cheng L. Kong C. Walvoort M. T. C. Faas M. M. De Vos P. (2020). Human milk oligosaccharides differently modulate goblet cells under homeostatic, proinflammatory conditions and ER stress. *Mol. Nutr. Food Res.* 64:1900976. 10.1002/mnfr.201900976 31800974 PMC7079026

[B10] Choi C. H. (2004). Prophylactic effect of *Lactobacillus* GG in animal colitis and its effect on cytokine secretion and mucin gene expressions. *Korean J. Gastroenterol.* 44 50–52. 15266134

[B11] Cilla A. Rodrigo M. J. Zacarías L. De Ancos B. Sánchez-Moreno C. Barberá R. (2018). Protective effect of bioaccessible fractions of citrus fruit pulps against H_2_O_2_-induced oxidative stress in Caco-2 cells. *Food Res. Int.* 103 335–344. 10.1016/j.foodres.2017.10.066 29389623

[B12] De Keersmaecker S. C. J. Varszegi C. Van Boxel N. Habel L. W. Metzger K. Daniels R. (2005). Chemical synthesis of (*S*)-4,5-Dihydroxy-2,3-pentanedione, a bacterial signal molecule precursor, and validation of its activity in *Salmonella* typhimurium*. *J. Biol. Chem.* 280 19563–19568. 10.1074/jbc.M412660200 15790567

[B13] Doron S. Gorbach S. L. (2006). Probiotics: their role in the treatment and prevention of disease. *Expert Rev. Ant Infect. Ther.* 4 261–275. 10.1586/14787210.4.2.261 16597207

[B14] Edgar R. C. Flyvbjerg H. (2015). Error filtering, pair assembly and error correction for next-generation sequencing reads. *Bioinformatics* 31 3476–3482. 10.1093/bioinformatics/btv401 26139637

[B15] Guo W. Zeng M. Zhu S. Li S. Qian Y. Wu H. (2022). Phycocyanin ameliorates mouse colitis via phycocyanobilin-dependent antioxidant and anti-inflammatory protection of the intestinal epithelial barrier. *Food Funct.* 13 3294–3307. 10.1039/D1FO02970C 35244658

[B16] Guo W. Zhu S. Feng G. Wu L. Feng Y. Guo T. (2020). Microalgae aqueous extracts exert intestinal protective effects in Caco-2 cells and dextran sodium sulphate-induced mouse colitis. *Food Funct.* 11 1098–1109. 10.1039/c9fo01028a 31825424

[B17] Hsiao A. Zhu J. (2020). Pathogenicity and virulence regulation of *Vibrio cholerae* at the interface of host-gut microbiome interactions. *Virulence* 11 1582–1599. 10.1080/21505594.2020.1845039 33172314 PMC7671094

[B18] Ichikawa-Tomikawa N. Sugimoto K. Satohisa S. Nishiura K. Chiba H. (2011). Possible involvement of tight junctions, extracellular matrix and nuclear receptors in epithelial differentiation. *J. Biomed. Biotechnol.* 2011:253048. 10.1155/2011/253048 22162632 PMC3227411

[B19] Ijssennagger N. Van Der Meer R. Van Mil S. W. C. (2016). Sulfide as a mucus barrier-breaker in inflammatory bowel disease? *Trends Mol. Med.* 22 190–199. 10.1016/j.molmed.2016.01.002 26852376

[B20] Ismail A. S. Valastyan J. S. Bassler B. L. (2016). A Host-Produced autoinducer-2 mimic activates bacterial quorum sensing. *Cell Host Microbe* 19 470–480. 10.1016/j.chom.2016.02.020 26996306 PMC4869860

[B21] Kaur A. Capalash N. Sharma P. (2020). Expression of *Meiothermus* ruber *luxS* in *E. coli* alters the antibiotic susceptibility and biofilm formation. *Appl. Microbiol. Biotechnol.* 104 4457–4469. 10.1007/s00253-020-10480-8 32215705

[B22] Kozich J. J. Westcott S. L. Baxter N. T. Highlander S. K. Schloss P. D. (2013). Development of a dual-index sequencing strategy and curation pipeline for analyzing amplicon sequence data on the MiSeq Illumina sequencing platform. *Appl. Environ. Microbiol.* 79 5112–5120. 10.1128/AEM.01043-13 23793624 PMC3753973

[B23] Laroui H. Ingersoll S. A. Liu H. C. Baker M. T. Ayyadurai S. Charania M. A. (2012). Dextran Sodium Sulfate (DSS) induces colitis in mice by forming nano-lipocomplexes with medium-chain-length fatty acids in the colon. *PLoS One* 7:e32084. 10.1371/journal.pone.0032084 22427817 PMC3302894

[B24] Lee J. Yun H. S. Cho K. W. Oh S. Kim S. H. Chun T. (2011). Evaluation of probiotic characteristics of newly isolated *Lactobacillus spp.*: immune modulation and longevity. *Int. J. Food Microbiol.* 148 80–86. 10.1016/j.ijfoodmicro.2011.05.003 21652104

[B25] Li J. Yang X. Shi G. Chang J. Liu Z. Zeng M. (2019). Cooperation of lactic acid bacteria regulated by the AI-2/LuxS system involve in the biopreservation of refrigerated shrimp. *Food Res. Int.* 120 679–687. 10.1016/j.foodres.2018.11.025 31000286

[B26] Li Q. Peng W. Wu J. Wang X. Ren Y. Li H. (2019). Autoinducer-2 of gut microbiota, a potential novel marker for human colorectal cancer, is associated with the activation of TNFSF9 signaling in macrophages. *Oncoimmunology* 8:e1626192. 10.1080/2162402X.2019.1626192 31646072 PMC6791418

[B27] Martens E. C. Neumann M. Desai M. S. (2018). Interactions of commensal and pathogenic microorganisms with the intestinal mucosal barrier. *Nat. Rev. Microbiol.* 16 457–470. 10.1038/s41579-018-0036-x 29904082

[B28] Mashimo H. Wu D.-C. Podolsky D. K. Fishman M. C. (1996). Impaired defense of intestinal mucosa in mice lacking intestinal trefoil factor. *Science* 274 262–265. 10.1126/science.274.5285.262 8824194

[B29] Matsuoka K. Kanai T. (2015). The gut microbiota and inflammatory bowel disease. *Semin. Immunopathol.* 37 47–55. 10.1007/s00281-014-0454-4 25420450 PMC4281375

[B30] Neurath M. F. (2014). Cytokines in inflammatory bowel disease. *Nat. Rev. Immunol.* 14 329–342. 10.1038/nri3661 24751956

[B31] Papanicolas L. E. Gordon D. L. Wesselingh S. L. Rogers G. B. (2020). Improving risk–benefit in faecal transplantation through microbiome screening. *Trends Microbiol.* 28 331–339. 10.1016/j.tim.2019.12.009 31952909

[B32] Pereira C. S. Thompson J. A. Xavier K. B. (2013). AI-2-mediated signalling in bacteria. *FEMS Microbiol. Rev.* 37 156–181. 10.1111/j.1574-6976.2012.00345.x 22712853

[B33] Petrella C. (2016). *Lactobacillus reuteri* treatment and DSS colitis: new insight into the mechanism of protection. *Acta Physiol.* 217 274–275. 10.1111/apha.12719 27228414

[B34] Qian Y. Li Y. Xu T. Zhao H. Zeng M. Liu Z. (2022). Dissecting of the AI-2/luxs mediated growth characteristics and bacteriostatic ability of *Lactiplantibacillus plantarum* SS-128 by integration of transcriptomics and metabolomics. *Foods* 11:638. 10.3390/foods11050638 35267271 PMC8909743

[B35] Quast C. Pruesse E. Yilmaz P. Gerken J. Schweer T. Yarza P. (2012). The SILVA ribosomal RNA gene database project: improved data processing and web-based tools. *Nucleic Acids Res.* 41 D590–D596. 10.1093/nar/gks1219 23193283 PMC3531112

[B36] Ranava D. Backes C. Karthikeyan G. Ouari O. Soric A. Guiral M. (2021). Metabolic exchange and energetic coupling between nutritionally stressed bacterial species: role of quorum-sensing molecules. *mBio* 12 e2758–e2820. 10.1128/mBio.02758-20 33468690 PMC7845633

[B37] Rodríguez-Nogales A. Algieri F. Garrido-Mesa J. Vezza T. Utrilla M. P. Chueca N. (2017). Differential intestinal anti-inflammatory effects of *Lactobacillus fermentum* and *Lactobacillus salivarius* in DSS mouse colitis: impact on microRNAs expression and microbiota composition. *Mol. Nutr. Food Res.* 61:1700144. 10.1002/mnfr.201700144 28752563

[B38] Schloss P. D. Gevers D. Westcott S. L. (2011). Reducing the effects of PCR amplification and sequencing artifacts on 16S rRNA-Based studies. *PLoS One* 6:e27310. 10.1371/journal.pone.0027310 22194782 PMC3237409

[B39] Schroeder B. O. Birchenough G. M. H. Ståhlman M. Arike L. Johansson M. E. V. Hansson G. C. (2018). Bifidobacteria or fiber protects against diet-induced microbiota-mediated colonic mucus deterioration. *Cell Host Microbe* 23 27.e7–40.e7. 10.1016/j.chom.2017.11.004 29276171 PMC5764785

[B40] Sokol H. Lepage P. Seksik P. Doré J. Marteau P. (2006). Temperature gradient gel electrophoresis of fecal 16S rRNA reveals active *Escherichia coli* in the microbiota of patients with ulcerative colitis. *J. Clin. Microbiol.* 44 3172–3177. 10.1128/JCM.02600-05 16954244 PMC1594675

[B41] Son S. J. Koh J. H. Park M. R. Ryu S. Lee W. J. Yun B. (2019). Effect of the *Lactobacillus rhamnosus* strain GG and tagatose as a synbiotic combination in a dextran sulfate sodium-induced colitis murine model. *J. Dairy Sci.* 102 2844–2853. 10.3168/jds.2018-15013 30799108

[B42] Soufli I. Toumi R. Rafa H. Touil-Boukoffa C. (2016). Overview of cytokines and nitric oxide involvement in immuno-pathogenesis of inflammatory bowel diseases. *World J. Gastrointest. Pharmacol. Ther.* 7:353–360. 10.4292/wjgpt.v7.i3.353 27602236 PMC4986402

[B43] Sun M. Liu Y. Song Y. Gao Y. Zhao F. Luo Y. (2020). The ameliorative effect of *Lactobacillus plantarum*-12 on DSS-induced murine colitis. *Food Funct.* 11 5205–5222. 10.1039/D0FO00007H 32458908

[B44] Taga M. E. Miller S. T. Bassler B. L. (2003). Lsr-mediated transport and processing of AI-2 in *Salmonella typhimurium*. *Mol. Microbiol.* 50 1411–1427. 10.1046/j.1365-2958.2003.03781.x 14622426

[B45] Thompson J. A. Oliveira R. A. Djukovic A. Ubeda C. Xavier K. B. (2015). Manipulation of the quorum sensing signal AI-2 affects the antibiotic-treated gut microbiota. *Cell Rep.* 10 1861–1871. 10.1016/j.celrep.2015.02.049 25801025

[B46] Tong L. Zhang X. Hao H. Liu Q. Zhou Z. Liang X. (2021). *Lactobacillus rhamnosus* GG Derived Extracellular vesicles modulate gut microbiota and attenuate inflammatory in DSS-Induced colitis mice. *Nutrients* 13:3319. 10.3390/nu13103319 34684320 PMC8541209

[B47] Wagner M. Peterson C. G. Ridefelt P. Sangfelt P. Carlson M. (2008). Fecal markers of inflammation used as surrogate markers for treatment outcome in relapsing inflammatory bowel disease. *World J. Gastroenterol.* 14 5584–5589. 10.3748/wjg.14.5584 18810778 PMC2746347

[B48] Wang G. Huang S. Cai S. Yu H. Wang Y. Zeng X. (2020). *Lactobacillus reuteri* ameliorates intestinal inflammation and modulates gut microbiota and metabolic disorders in dextran sulfate sodium-induced colitis in mice. *Nutrients* 12:2298. 10.3390/nu12082298 32751784 PMC7468961

[B49] Wine E. Ossa J. C. Gray-Owen S. D. Sherman P. (2010). Adherent-invasive *Escherichia coli* target the epithelial barrier. *Gut Microbes* 1 80–84. 10.4161/gmic.1.2.11142 21326914 PMC3023584

[B50] Winzer K. Hardie K. Williams P. (2003). LuxS and autoinducer-2: their contribution to quorum. *Adv. Appl. Microbiol.* 53:291. 10.1016/s0065-2164(03)53009-x 14696323

[B51] Wirtz S. Popp V. Kindermann M. Gerlach K. Weigmann B. Fichtner-Feigl S. (2017). Chemically induced mouse models of acute and chronic intestinal inflammation. *Nat. Protoc.* 12 1295–1309. 10.1038/nprot.2017.044 28569761

[B52] Wrzosek L. Miquel S. Noordine M.-L. Bouet S. Chevalier-Curt M. J. Robert V. (2013). *Bacteroides* thetaiotaomicron and *Faecalibacterium prausnitziiinfluence* the production of mucus glycans and the development of goblet cells in the colonic epithelium of a gnotobiotic model rodent. *BMC Biol.* 11:61. 10.1186/1741-7007-11-61 23692866 PMC3673873

[B53] Xavier K. B. Miller S. T. Lu W. Kim J. H. Rabinowitz J. Pelczer I. (2007). Phosphorylation and processing of the quorum-sensing molecule autoinducer-2 in enteric bacteria. *ACS Chem. Biol.* 2 128–136. 10.1021/cb600444h 17274596

[B54] Xu H. Hiraishi K. Kurahara L.-H. Nakano-Narusawa Y. Li X. Hu Y. (2021). Inhibitory effects of breast milk-derived *Lactobacillus rhamnosus* Probio-M9 on colitis-associated carcinogenesis by restoration of the gut microbiota in a mouse model. *Nutrients* 13:1143. 10.3390/nu13041143 33808480 PMC8065529

[B55] Zhao B. Xia B. Li X. Zhang L. Liu X. Shi R. (2020). Sesamol supplementation attenuates DSS-Induced colitis via mediating gut barrier integrity, inflammatory responses, and reshaping gut microbiome. *J. Agric. Food Chem.* 68 10697–10708. 10.1021/acs.jafc.0c04370 32893621

