# Supplementary material for: Amelioration of dextran sulfate sodium-induced colitis by autoinducer-2-deficient Lactiplantibacillus plantarum is mediated by anti-inflammatory effects and alleviation of dysbiosis of the gut microbiota
Source: Front Microbiol. 2022 Sep 14;13:1013586. doi: 10.3389/fmicb.2022.1013586 (PMC9515423; doi:10.3389/fmicb.2022.1013586)
Supplement: Supplementary file 1 [file Data_Sheet_1.docx]

Supplementary Material

## Supplementary Table 1. The criteria for DAI

| Score | Weight loss | Stool consistency | Rectal bleeding |
| --- | --- | --- | --- |
| 0 | None | Normal stools | Negative |
| 1 | 1-5% | Soft stools | Negative |
| 2 | 6-10% | Soft stools | Positive |
| 3 | 11-15% | Very soft | Visible in stool |
| 4 | > 15% | Watery stool | Gross bleeding |


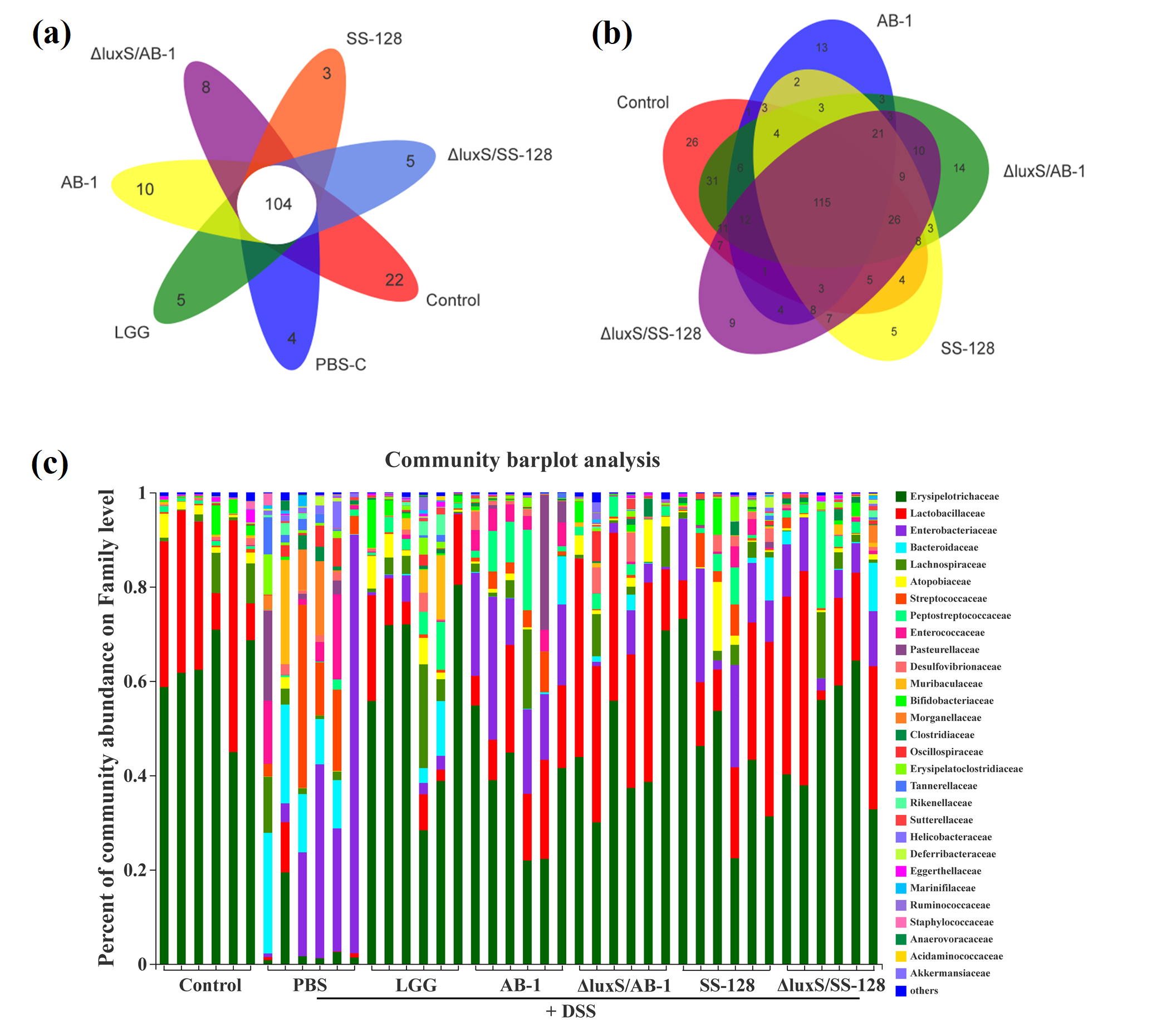
**Supplementary Figure 1.** Analysis of bacterial diversity in each group of mice following DSS administration: (a) Venn diagram showing the overlap of the OTUs identified in the intestinal microbiota among the normal control, DSS and LAB groups, (b) Venn diagram showing the overlap of the OTUs identified in the intestinal microbiota among the LAB groups and (c) At the family level.


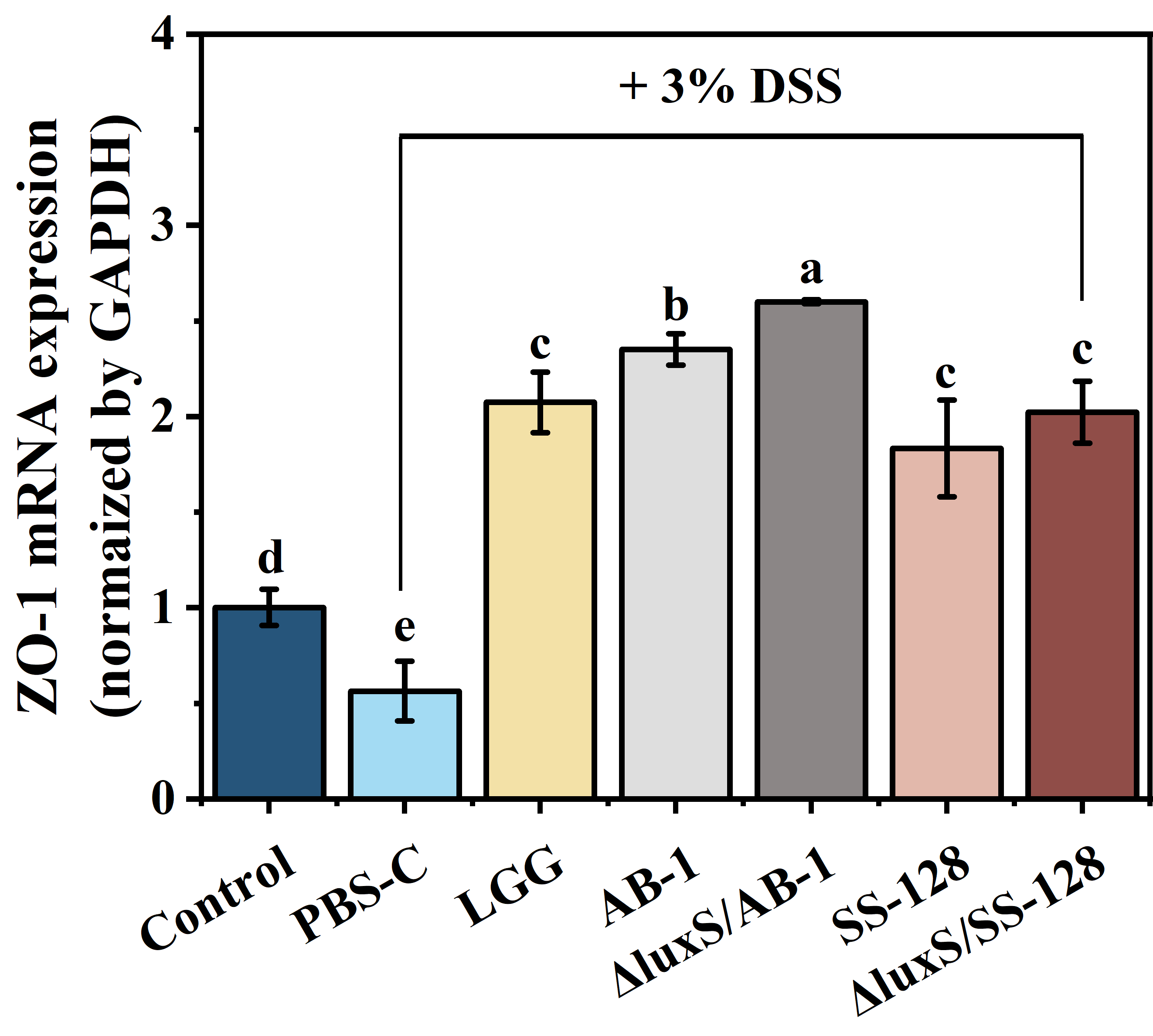


**Supplementary Figure 2.** mRNA expression of ZO-1. Data were expressed as means standard deviations (n = 6). Different superscript letters (a-e) denote statistically significant differences (*P* < 0.05).
